# Body mass index and survival in people with heart failure

**DOI:** 10.1136/heartjnl-2023-322459

**Published:** 2023-06-08

**Authors:** Nicholas R Jones, José M Ordóñez-Mena, Andrea K Roalfe, Kathryn S Taylor, Clare R Goyder, FD Richard Hobbs, Clare J Taylor

**Affiliations:** 1 Nuffield Department of Primary Care Health Sciences, University of Oxford, Oxford, UK; 2 NIHR Oxford Biomedical Research Centre, Oxford, UK

**Keywords:** Heart failure, Epidemiology, Obesity

## Abstract

**Aims:**

In people with heart failure (HF), a high body mass index (BMI) has been linked with better outcomes (‘obesity paradox’), but there is limited evidence in community populations across long-term follow-up. We aimed to examine the association between BMI and long-term survival in patients with HF in a large primary care cohort.

**Methods:**

We included patients with incident HF aged ≥45 years from the Clinical Practice Research Datalink (2000–2017). We used Kaplan-Meier curves, Cox regression and penalised spline methods to assess the association of pre-diagnostic BMI, based on WHO classification, with all-cause mortality.

**Results:**

There were 47 531 participants with HF (median age 78.0 years (IQR 70–84), 45.8% female, 79.0% white ethnicity, median BMI 27.1 (IQR 23.9–31.0)) and 25 013 (52.6%) died during follow-up. Compared with healthy weight, people with overweight (HR 0.78, 95% CI 0.75 to 0.81, risk difference (RD) −4.1%), obesity class I (HR 0.76, 95% CI 0.73 to 0.80, RD −4.5%) and class II (HR 0.76, 95% CI 0.71 to 0.81, RD −4.5%) were at decreased risk of death, whereas people with underweight were at increased risk (HR 1.59, 95% CI 1.45 to 1.75, RD 11.2%). In those underweight, this risk was greater among men than women (p value for interaction=0.02). Class III obesity was associated with increased risk of all-cause mortality compared with overweight (HR 1.23, 95% CI 1.17 to 1.29).

**Conclusion:**

The U-shaped relationship between BMI and long-term all-cause mortality suggests a personalised approach to identifying optimal weight may be needed for patients with HF in primary care. Underweight people have the poorest prognosis and should be recognised as high-risk.

**WHAT IS ALREADY KNOWN ON THIS TOPIC**

- Previous research suggests a so-called ‘obesity paradox’ exists, whereby people with obesity are more likely to develop heart failure (HF) but survive longer with it, compared with people with healthy weight. However, these data are based on clinical trial or secondary care studies and may not be generalisable to the wider population with HF seen in primary care, who are typically older and frailer.

**WHAT THIS STUDY ADDS**

- In this large primary care study, people with HF who were underweight were at the greatest risk of death, but only where body mass index (BMI) was recorded within 3 years of death or end of follow-up. In contrast, people with class I or II obesity were at lower risk of death than people with a healthy weight.

**HOW THIS STUDY MIGHT AFFECT RESEARCH, PRACTICE OR POLICY**

- Clinicians may need to adopt a tailored approach to discussions of weight management in people with HF, recognising that previous research suggests weight loss in those with obesity may improve symptoms but that a lower BMI is associated with worse survival. Future research could investigate the effect of intentional weight loss in terms of survival among people with HF.

## Introduction

Heart failure (HF) affects around 1%–2% of adults globally.^1^ The mean 5-year survival for people with HF is around 50%.^2^ Survival rates have shown only modest improvement since the start of the century, despite the discovery of several treatments that confer prognostic benefit.^3^

The prevalence of obesity has nearly tripled since 1975, with close to 2 billion adults globally now living with overweight or obesity.^4^ People with obesity are at increased risk of cardiovascular disease (CVD) and up to twice as likely to develop HF compared with people with a healthy body mass index (BMI).^5 6^ This association has been confirmed using other measures of adiposity, such as waist circumference and waist-hip ratio.^7^

Among people with established HF, increasing BMI has been linked to a reduced risk of all-cause mortality, the so-called ‘obesity paradox’.^8 9^ However, these data are from clinical trial participants or studies conducted in secondary or tertiary centres, meaning the results may not be generalisable to the larger population living with HF in the community.

We are not aware of any studies that have analysed the prognostic significance of BMI among people with HF using routine primary care data. Many patients will first present with symptoms of HF to primary care clinicians and an awareness of the impact of BMI on outcome could be important to guide clinical care. In this analysis, we examine the association between BMI and long-term survival in patients with HF in a large primary care cohort.

## Methods

The present study is a secondary analysis of the data obtained for ‘SurviveHF’, a retrospective electronic health record (EHR) linkage community study that compared long-term survival between people with and without HF.^3^

### Patient and public involvement

Two patients with HF informed the original ‘SurviveHF’ research question and study design. Their experience was that prognosis in HF was not often discussed and they had been surprised to find it was a long-term condition, rather than a terminal diagnosis. They wanted ‘doctors to have the facts’ about survival rates in HF to better inform discussions with patients.

### Design and setting

We completed a retrospective cohort study using routinely collected primary care data from Clinical Practice Research Datalink (CPRD), between 2000 and 2017. Participating practices contributed to at least 1 year of data and met data quality measures. The data are linked to secondary care Hospital Episode Statistics (HES) to help ensure the accuracy of diagnostic codes. This limited the study to practices in England. Data are also linked to Office for National Statistics (ONS) mortality data and the Index of Multiple Deprivation (IMD) socio-economic data.

### Study population

We included patients aged ≥45 years who had an incident diagnostic code for HF in their EHR during the study period, were eligible for data linkage and registered at an eligible practice for ≥12 months. We excluded patients with no recorded BMI or diagnosed with HF prior to 1 January 2000.

Patients entered the cohort at the latest of the following: their 45th birthday, 1 January 2000, patient registration date plus 12 months, practice up-to-standard date plus 12 months. Patients exited the cohort on the earliest of the following: 31 December 2017, date of death, patient transfer out date, last date of practice data collection, last date of available linked data.

### Case definition

We defined new HF cases as the earliest recorded diagnostic code in the EHR (the index date). Time to death was measured from the index date.

### Exposure assessment

BMI was categorised according to the WHO classification as underweight (BMI<18.5 kg/m^2^), healthy weight (18.5–24.9 kg/m^2^), overweight (25–29.9 kg/m^2^) or obesity class I (30–34.9 kg/m^2^), II (35–39.9 kg/m^2^) or III (≥40 kg/m^2^).^10^ We used the most recent BMI reading on or before the index date. Recent data support a move to more ethnicity-specific BMI cut-offs,^11^ so we also categorised BMI into finer data-driven categories using deciles to assess the shape of the relationship with all-cause mortality.

### Covariates

Patient-level characteristics included age, sex, ethnicity, smoking status, IMD, systolic and diastolic blood pressure, total cholesterol, and history of atrial fibrillation, angina, ischaemic heart disease, myocardial infarction, hypertension, type 2 diabetes mellitus, stroke, valvular disease and other CVDs.

### Outcomes

The primary outcome was all-cause mortality. Secondary outcomes included cause of death and cardiovascular mortality. We also assessed effect modification by sex of the association of BMI with all-cause mortality.

### Statistical analysis

We undertook a descriptive analysis of the cohort based on categories of BMI. We calculated median time and IQR between BMI measurement and HF diagnosis. Causes of death were tabulated by BMI categories. Kaplan-Meier survival curves were produced to compare BMI categories and to estimate 1-year, 5-year, 10-year and 15-year overall survival rates by BMI subgroups as a univariable analysis in the whole cohort and within age categories. Cox regression was used to predict age-adjusted survival rates by BMI category.

For the primary analysis, we ran a Cox proportional hazards model to estimate HRs and 95% CIs, as a univariable analysis, age and sex adjusted and then a multivariable model adjusted for the covariates listed above. Penalised splines were used to model BMI as a continuous variable and assess whether the relationship with all-cause mortality was non-linear. Cox models were stratified by sex to assess variability in the association of BMI with all-cause mortality, and interaction terms of BMI categories with sex and ethnicity were added to the primary analysis to test for effect modification.

Low BMI close to the end of life may identify people with a poor prognosis because of factors such as sarcopenia, cachexia or advanced frailty. High BMI in people with HF may be due to fluid overload and therefore sometimes reflects more advanced disease. To discard the influence of reverse causality leading to incorrect conclusions about the direction of association between BMI and risk of death, a sensitivity analysis was conducted repeating the primary analysis after excluding deaths occurring within 1, 2, 3, 4 and 5 years of BMI measurement.

We suspected data such as BMI, smoking and cholesterol would not be missing at random and so chose to use a complete case analysis, as multiple imputation may lead to biased estimates.^12^ We performed multiple imputation with chained equations to confirm the results of the main analysis were not significantly different to the complete case analysis.

All analyses were completed in R V.4.2.0 (Vienna, Austria).^13 14^

## Results

There were 47 531 included patients with a median age of 78.0 years (IQR 70–84) and BMI 27.1 (IQR 23.9–31.0). The median time between BMI measurement and HF diagnosis was 1 year (IQR 0.33–3 years). Most participants were overweight (36.3%) compared with 31.3% having a healthy weight, 30.1% having obesity (19.0%, 7.2% and 3.9% with obesity classes I, II and III, respectively) and 2.3% underweight.

People with a higher than healthy BMI were more likely to be younger and current smoker and have type 2 diabetes and hypertension, whereas people with underweight were more likely to be female, older and have a history of stroke (table 1).

**Table 1:** Baseline characteristics of people with heart failure stratified by BMI category

Variable	Underweight		Healthy weight		Overweight		Obesity class I		Obesity class II		Obesity class III		All cohort	
	(<18.5 kg/m^2^)		(18.5–25 kg/m^2^)		(25–30 kg/m^2^)		(30–35 kg/m^2^)		(35–40 kg/m^2^)		(≥40 kg/m^2^)			
Total	1084	(100)	14 880	(100)	17 277	(100)	9013	(100)	3400	(100)	1877	(100)	47 531	(100)
Gender, n (%)														
Men	315	(29.06)	7429	(49.93)	10 486	(60.69)	5086	(56.43)	1674	(49.24)	765	(40.76)	25 755	(54.19)
Women	769	(70.94)	7451	(50.07)	6791	(39.31)	3927	(43.57)	1726	(50.76)	1112	(59.24)	21 776	(45.81)
Age (years), median (IQR)	82	(75–87)	81	(74–86)	78	(71–84)	76	(68–82)	73	(65–79)	68	(60–75)	78	(70–84)
Age category, n (%)														
45–54	17	(1.57)	316	(2.12)	542	(3.14)	390	(4.33)	210	(6.18)	189	(10.07)	1664	(3.50)
55–64	64	(5.90)	980	(6.59)	1497	(8.66)	1140	(12.65)	609	(17.91)	519	(27.65)	4809	(10.12)
65–74	165	(15.22)	2624	(17.63)	4002	(23.16)	2605	(28.90)	1139	(33.50)	654	(34.84)	11 189	(23.54)
75–84	416	(38.38)	5952	(40.00)	7157	(41.43)	3492	(38.74)	1138	(33.47)	441	(23.49)	18 596	(39.12)
85–94	391	(36.07)	4620	(31.05)	3870	(22.40)	1328	(14.73)	291	(8.56)	74	(3.94)	10 574	(22.25)
94+	31	(2.86)	388	(2.61)	209	(1.21)	58	(0.64)	13	(0.38)	0	(0.00)	699	(1.47)
Ethnicity, n (%)														
White	863	(79.61)	11 755	(79.00)	13 731	(79.48)	7018	(77.87)	2696	(79.29)	1474	(78.53)	37 537	(78.97)
Non-white	16	(1.48)	381	(2.56)	489	(2.83)	296	(3.28)	110	(3.24)	56	(2.98)	1348	(2.84)
Mixed	127	(11.72)	1738	(11.68)	2155	(12.47)	1288	(14.29)	490	(14.41)	280	(14.92)	6078	(12.79)
Missing	78	(7.20)	1006	(6.76)	902	(5.22)	411	(4.56)	104	(3.06)	67	(3.57)	2568	(5.40)
Smoking status, n (%)														
Never	403	(37.18)	6137	(41.24)	6554	(37.93)	3172	(35.19)	1191	(35.03)	649	(34.58)	18 106	(38.09)
Former	260	(23.99)	2240	(15.05)	2013	(11.65)	998	(11.07)	398	(11.71)	243	(12.95)	6152	(12.94)
Current	406	(37.45)	6337	(42.59)	8531	(49.38)	4746	(52.66)	1771	(52.09)	960	(51.15)	22 751	(47.87)
Missing	15	(1.38)	166	(1.12)	179	(1.04)	97	(1.08)	40	(1.18)	25	(1.33)	522	(1.10)
Index of multiple deprivation (quintile), n (%)														
1 (most deprived)	181	(16.70)	3191	(21.44)	3534	(20.45)	1570	(17.42)	530	(15.59)	238	(12.68)	9244	(19.45)
2	260	(23.99)	3522	(23.67)	4052	(23.45)	1970	(21.86)	699	(20.56)	351	(18.70)	10 854	(22.84)
3	230	(21.22)	3111	(20.91)	3723	(21.55)	1957	(21.71)	755	(22.21)	401	(21.36)	10 177	(21.41)
4	236	(21.77)	2990	(20.09)	3539	(20.48)	2001	(22.20)	776	(22.82)	496	(26.43)	10 038	(21.12)
5 (least deprived)	175	(16.14)	2048	(13.76)	2415	(13.98)	1507	(16.72)	640	(18.82)	390	(20.78)	7175	(15.10)
Missing	2	(0.18)	18	(0.12)	14	(0.08)	8	(0.09)	0	(0.00)	1	(0.05)	43	(0.09)
Medical history of														
Atrial fibrillation, n (%)	280	(25.83)	4195	(28.19)	4748	(27.48)	2393	(26.55)	894	(26.29)	470	(25.04)	12 980	(27.31)
Angina, n (%)	175	(16.14)	3214	(21.60)	4160	(24.08)	2201	(24.42)	765	(22.50)	356	(18.97)	10 871	(22.87)
Type 2 diabetes mellitus, n (%)	132	(12.18)	2480	(16.67)	4251	(24.60)	3056	(33.91)	1502	(44.18)	1004	(53.49)	12 425	(26.14)
Hypertension, n (%)	501	(46.22)	8122	(54.58)	10 512	(60.84)	6057	(67.20)	2474	(72.76)	1386	(73.84)	29 052	(61.12)
Ischaemic heart disease, n (%)	222	(20.48)	3934	(26.44)	5095	(29.49)	2664	(29.56)	870	(25.59)	411	(21.90)	13 196	(27.76)
Myocardial infarction, n (%)	186	(17.16)	3095	(20.80)	4043	(23.40)	2048	(22.72)	635	(18.68)	264	(14.06)	10 271	(21.61)
Other cardiovascular disease, n (%)	288	(26.57)	3944	(26.51)	4563	(26.41)	2219	(24.62)	771	(22.68)	381	(20.30)	12 166	(25.60)
Stroke, n (%)	131	(12.08)	1759	(11.82)	2040	(11.81)	1024	(11.36)	339	(9.97)	139	(7.41)	5432	(11.43)
Valvular disease, n (%)	101	(9.32)	1407	(9.46)	1411	(8.17)	559	(6.20)	180	(5.29)	78	(4.16)	3736	(7.86)
BMI (kg/m^2^), median (IQR)	17.54	(16.8–18.04)	22.8	(21.27–23.99)	27.29	(26.14–28.53)	31.97	(30.9–33.23)	36.91	(35.85–38.11)	43.71	(41.49–47.33)	27.06	(23.88–31)
Systolic blood pressure (mm Hg), median (IQR)	132	(120–146)	136	(120–148)	137	(124–150)	138	(125–150)	140	(127–150)	138.5	(126–150)	137	(123–150)
Missing	7	(0.65)	83	(0.56)	77	(0.45)	37	(0.41)	5	(0.15)	3	(0.16)	212	(0.45)
Diastolic blood pressure (mm Hg), median (IQR)	74	(67–80)	76	(70–82)	78	(70–82)	79	(70–84)	80	(70–85)	80	(70–86)	78	(70–83)
Missing	7	(0.65)	83	(0.56)	77	(0.45)	37	(0.41)	5	(0.15)	3	(0.16)	212	(0.45)
Total cholesterol (mmol/L), median (IQR)	4.7	(3.95–5.5)	4.6	(3.8–5.5)	4.5	(3.8–5.4)	4.5	(3.8–5.3)	4.5	(3.8–5.3)	4.4	(3.7–5.2)	4.5	(3.8–5.4)
Missing	373	(34.41)	4082	(27.43)	3623	(20.97)	1462	(16.22)	398	(11.71)	202	(10.76)	10 140	(21.33)

BMI, body mass index.

For this analysis, 8428 (15.1%) were removed from the original cohort because they did not have a recorded BMI in their EHR. These people were more likely to be women, older and to have missing information on ethnicity, smoking status and medical history (online supplemental table 1).

10.1136/heartjnl-2023-322459.supp1 Supplementary data

### Risk of all-cause mortality

**Figure 1:**
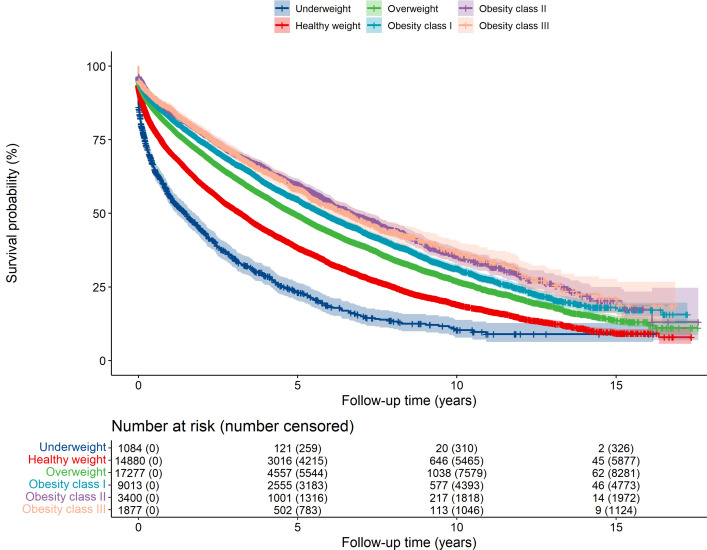
Survival over time for people with heart failure, according to their body mass index. The Kaplan-Meier curve shows that the probability of survival is lowest among people with heart failure who are underweight, then people with healthy weight compared with people with overweight or obesity.

There were 25 013 deaths during a mean 3.3 years of follow-up. The 1-year, 5-year, 10-year and 15-year overall survival rates appeared to improve from lower to higher BMI groups (table 2). For example, the 10-year survival rate among people with underweight was 10.3% (95% CI 7.8% to 13.8%) compared with 19.1% (95% CI 18.1% to 20.1%) with healthy weight and 31.2% (95% CI 29.7% to 32.8%) with class I obesity. This pattern was also seen after stratifying and adjusting by age (online supplemental tables 2 and 3). Figure 1 shows the lower probability of overall survival over time among people with underweight, healthy weight or overweight compared with those with obesity.

**Table 2:** Survival rates at 1, 5, 10 and 15 years after a diagnosis of HF stratified by BMI category

Subgroup	Survival rate (% (95% CI))			
	1 year	5 years	10 years	15 years
Overall*	77.45 (77.07 to 77.84)	47.42 (46.90 to 47.95)	25.83 (25.22 to 26.45)	13.57 (12.70 to 14.50)
By BMI category:				
Underweight (<18.5 kg/m^2^)	55.85 (52.89 to 58.98)	23.14 (20.28 to 26.40)	10.33 (7.75 to 13.77)	8.98 (6.34 to 12.71)
Healthy weight (18.5–25 kg/m^2^)	70.80 (70.06 to 71.55)	38.39 (37.48 to 39.32)	19.07 (18.13 to 20.07)	9.20 (7.96 to 10.64)
Overweight (25–30 kg/m^2^)	79.52 (78.90 to 80.13)	49.35 (48.48 to 50.23)	26.95 (25.94 to 27.99)	13.58 (12.09 to 15.26)
Obesity class I (30–35 kg/m^2^)	82.60 (81.80 to 83.40)	54.75 (53.55 to 55.98)	31.22 (29.73 to 32.78)	18.09 (16.15 to 20.27)
Obesity class II (35–40 kg/m^2^)	84.66 (83.43 to 85.91)	59.87 (57.96 to 61.86)	35.56 (33.02 to 38.29)	20.31 (16.53 to 24.96)
Obesity class III (≥40 kg/m^2^)	85.83 (84.23 to 87.46)	58.19 (55.51 to 61.01)	36.84 (33.34 to 40.71)	20.99 (15.93 to 27.66)

*Among people with HF and a measurement of BMI.

BMI, body mass index; HF, heart failure.

After adjusting for potential confounders, compared with people with healthy weight, people with underweight were at increased risk of all-cause mortality (HR 1.59, 95% CI 1.45 to 1.75, risk difference (RD) 11.2%). People with overweight (HR 0.78, 95% CI 0.75 to 0.81, RD −4.1%), obesity class I (HR 0.76, 95% CI 0.73 to 0.80, RD −4.5%) and obesity class II (HR 0.76, 95% CI 0.71 to 0.81, RD −4.5%) were at decreased risk of all-cause mortality (table 3, online supplemental table 4). People with obesity class III were not at increased risk of all-cause mortality compared with those with healthy weight, but were at greater risk than those with overweight or obesity class I or II. The HR for death comparing people with obesity class III to overweight was 1.23 (95% CI 1.17 to 1.29). The increased risk of all-cause mortality with underweight was greater among men than women (p value for interaction=0.02) (online supplemental table 5). There was no evidence of effect modification by sex for the higher BMI categories.

**Table 3:** HRs and 95% CIs for the association of BMI categories with all-cause mortality among people with heart failure

BMI category	N	n	Crude model		Age and sex adjusted		N*	n*	Multivariable model†	
Underweight (<18.5 kg/m^2^)	1084	756	1.59	(1.48 to 1.71)	1.52	(1.41 to 1.64)	681	446	1.59	(1.45 to 1.75)
Healthy weight (18.5–25 kg/m^2^)	14 880	8959	1.00	(Reference)	1.00	(Reference)	10 223	5579	1.00	(Reference)
Overweight (25–30 kg/m^2^)	17 277	8939	0.74	(0.72 to 0.76)	0.81	(0.79 to 0.84)	13 071	6054	0.78	(0.75 to 0.81)
Obesity class I (30–35 kg/m^2^)	9013	4197	0.64	(0.62 to 0.66)	0.79	(0.76 to 0.82)	7235	3045	0.76	(0.73 to 0.80)
Obesity class II (35–40 kg/m^2^)	3400	1417	0.56	(0.53 to 0.59)	0.78	(0.74 to 0.82)	2904	1123	0.76	(0.71 to 0.81)
Obesity class III (≥40 kg/m^2^)	1877	745	0.56	(0.52 to 0.60)	0.95	(0.88 to 1.02)	1614	594	0.96	(0.88 to 1.05)

*The numbers at risk and dying are smaller in the multivariable model due to missing data.

†Adjusted for sex, age, smoking status, index of multiple deprivation, ethnicity, systolic and diastolic blood pressure, total cholesterol, and history of atrial fibrillation, angina, type 2 diabetes mellitus, hypertension, ischaemic heart disease, myocardial infarction, stroke, valvular heart disease and other cardiovascular diseases.

BMI, body mass index; N, number at risk of dying within the relevant BMI category; n, number dying from all-causes for the BMI category.

Testing for an interaction between ethnicity and BMI found no significant difference from our main findings (online supplemental table 6). The results of the complete case analysis and multiple imputation were broadly similar (online supplemental table 7). We confirmed that the proportional hazards assumption was met using Schoenfield’s residual plots.

**Figure 2:**
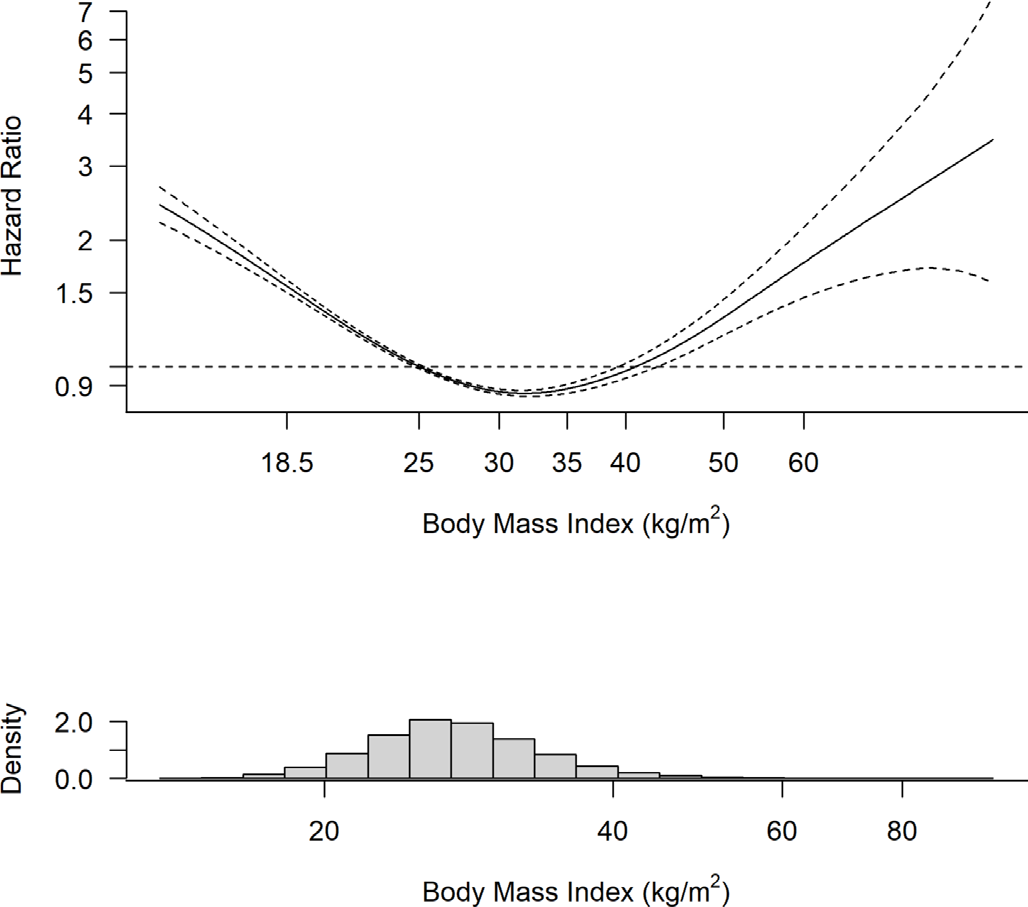
Relationship between BMI and all-cause mortality among people with heart failure. The modelled penalised spline regression demonstrates a U-shaped relationship between BMI and risk of all-cause mortality among people with heart failure. Dotted lines denote the 95% CIs. BMI, body mass index.

**Figure 3:**
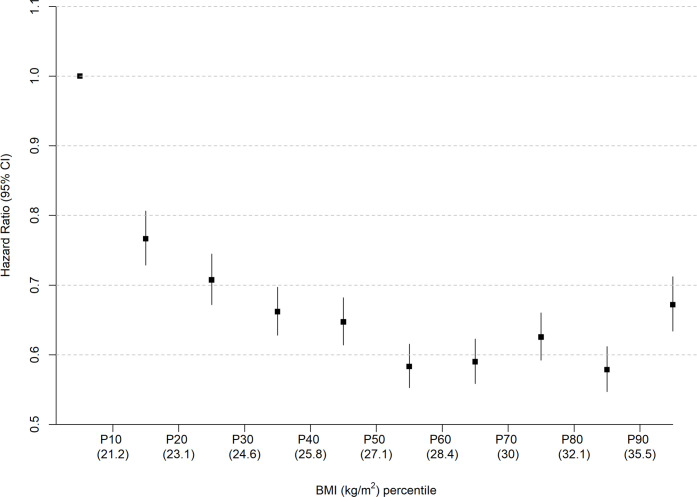
Association of BMI categories by deciles with all-cause mortality. HR and 95% CI for the association between deciles of BMI category and all-cause mortality among people with heart failure. People with overweight or class I obesity are at lowest risk, while people in the lowest weight decile have the highest risk of death. BMI, body mass index.

The analysis using penalised splines showed a U-shaped relationship whereby those with overweight and obesity class I were at lowest risk of all-cause mortality (figure 2). The analysis using deciles of equally spaced BMI categories also showed no evidence of a linear inverse association between BMI and all-cause mortality (figure 3).

The increased risk of all-cause mortality among people with underweight was sensitive to the time between BMI assessment and death (online supplemental table 8). The association was no longer there in people who had their BMI measurement recorded more than 3 years apart from their death or end of follow-up. The same principle applied to the decreased risks of all-cause mortality seen among those with obesity classes I and II. In contrast, the lower risk of death seen among people with HF who were overweight was robust to the exclusions of those with their BMI assessment within 5 years of their time of death or censoring.

### Cause-specific mortality

Among people who were underweight, a relatively lower proportion died from CVD or cancer and a higher proportion died from respiratory diseases compared with other weight categories (online supplemental table 9). However, fewer people with overweight or obesity died directly from HF or had HF listed as a contributory cause of death compared with people with underweight or obesity class III, meaning the higher CVD mortality in these two groups was due to cardiovascular conditions other than HF.

After adjusting for potential confounders, people who were underweight were at increased risk of dying from CVD compared with people with healthy weight (HR 1.51, 95% CI 1.32 to 1.73) (online supplemental table 10). People with overweight (HR 0.79, 95% CI 0.75 to 0.83), obesity class I (HR 0.77, 95% CI 0.72 to 0.81) and obesity class II (HR 0.78, 95% CI 0.71 to 0.85) were at decreased risk of dying from CVD.

## Discussion

In this large primary care cohort of people with HF, those with underweight were at the highest risk of all-cause and cardiovascular mortality. People with overweight, class I or II obesity were at a decreased relative risk of death and HF-related mortality. Our results show that the obesity paradox endures over longer-term follow-up and among patients with HF in community settings.

### Comparison with existing literature

Previous systematic reviews consistently report that people with HF and obesity or overweight were at decreased risk of all-cause and cardiovascular mortality compared with healthy weight.^6 15 16^ However, these studies rely on data from secondary care or trial participants, typically with relatively short-term follow-up and younger participants (mean age 54–72 years) than our real-world primary care study (76.6 years).^16 17^ We found that a similar association is seen over extended follow-up among older people with chronic HF. The previous reviews have found only limited data with regard to cardiovascular mortality in people with underweight.^6^

Our age-specific results showed that survival rates were lower for people who were underweight, irrespective of age (online supplemental table 3). In contrast, an individual patient data analysis including 6000 patients with chronic HF reported that the increased risk of all-cause mortality among people with underweight or healthy BMI was only seen in patients aged ≥75 years with at least one co-morbidity.^18^ The authors concluded that lower BMI may be a marker of advanced HF or cardiac cachexia and a poor prognostic indicator in older people only.^18^ This study relied on patients in clinical trials with a younger mean age (65 years (SD 12)) than is typical in HF. In contrast, our cohort was older and the inverse relationship seen between weight status and survival is likely to be generalisable to the majority of patients with HF.

### Strengths and limitations

To our knowledge, this is the first study conducted in primary care to report long-term survival rates among people with HF in relation to their BMI. Our analysis draws on a large cohort of patients and is likely to be broadly representative of people with HF living in high-income countries.

We chose to use the most recent BMI prior to HF diagnosis because this provided a time point that was comparable for all patients in their disease course. However, height and weight are inconsistently recorded in primary care with a clustering of weight recordings expected in women, younger and older adults, those with low or high BMI and in people with relevant comorbid health conditions.^19^ Missing data may not have been missing at random and people with healthy weight may be under-represented.

BMI has been criticised as a poor measure of weight status as it does not differentiate between adipose tissue, lean muscle mass or weight gain due to oedema, which may be of particular importance in a population with HF. A recent UK Biobank study found that low BMI (<18.5 kg/m^2^) was associated with an increased risk of incident CVD compared with a log-linear association for other measures of adiposity, such as waist circumference or percentage body fat mass.^20^ The authors suggest that this may be because the association between health status and BMI is more prone to confounding or reverse causality.^20^ Nonetheless, BMI is widely seen as a reliable marker of percentage body fat across populations and alternative measures, such as waist circumference, are rarely coded in primary care.^21^ We tested for the existence of reverse causality by repeating the primary analysis after excluding deaths occurring close to the time of BMI measurement. Underweight was only associated with an increased risk of death if the most recent BMI measurement was made within 2 years of death, suggesting that cachexia at the end of life is important rather than underweight in earlier life. However, there were only small numbers of participants in these subgroups and it is difficult to draw definite conclusions. In contrast, the reduced risk of death in people with overweight or obesity class I was robust to the time between weight measurement and death.

Data were only available on the most recent BMI measure and so we were not able to analyse changes in BMI, nor the duration of overweight or obesity, even though these factors may impact on the risk of mortality. Misclassification by BMI is also possible in participants whose weight changed significantly over time. Residual confounding related to factors not well coded in the EHR cannot be excluded, such as cardiorespiratory fitness levels.^22^ Results are not adjusted for treatment effect or aetiology of HF, though the prognostic significance of these factors are not known to be altered by weight status.^23^

The recording of HF in primary care rarely categorises the left ventricular ejection fraction, and it was therefore not possible to explore the relevance of this in relation to survival. This may be important as increasing BMI is associated with a greater risk of developing heart failure with preserved ejection fraction (HFpEF) as opposed to heart failure with reduced ejection fraction (HFrEF),^9^ though an association between reduced mortality and increased body weight has been reported among populations with both HFrEF^24^ and HFpEF.^25^

### Implications for practice and future research

The mechanism of the obesity paradox remains unclear. It has been postulated that people with obesity may benefit from greater metabolic reserves or an attenuated response of the renin-angiotensin-aldosterone system.^8^ People with obesity typically have higher arterial blood pressure, which might mean higher doses of medications are tolerated that confer a prognostic benefit in HF.^8^

Clinicians may need to adopt a tailored approach to discussing weight management in HF, accounting for baseline BMI. Previous research suggests that the obesity paradox only exists among people with poor cardiorespiratory fitness,^8 26^ and small amounts of weight loss and improvements in fitness levels may improve outcomes in HF.^27^ Weight management programmes for people with HFpEF and obesity have been shown to improve exercise capacity and overall quality of life.^28^ Furthermore, increasing BMI is independently associated with risk of HF hospitalisation among people with HFpEF.^29^ There remains a need for further research into the role of intentional weight loss in people with HF to determine the impact this may have on symptoms and quality of life as well as survival.^27^

Clinicians should recognise that cachexia and low BMI, particularly in the context of clinical frailty, are likely to be markers of poor prognosis in people with HF. Prevalence of frailty is far higher among people with HF compared with the age-matched population, and the presence of frailty and HF are associated with an 80% higher risk of all-cause mortality.^30^ A multidisciplinary approach including physical rehabilitation and support for diet and nutrition may be helpful for this cohort of patients.

## Conclusions

In this large, long-term primary care cohort study, we observed a U-shaped relationship between BMI and HF mortality. People with overweight and class I or II obesity were at the lowest risk, supporting previous findings of an ‘obesity paradox’ for HF survival in relation to body weight. In contrast, the relatively small number of people with underweight were at the greatest risk of death and should be identified as such by clinicians. Further research is needed to inform recommendations around the possible benefits of intentional weight loss for patients with HF.

## Data Availability

Data may be obtained from a third party and are not publicly available. Data for this study were obtained from the Clinical Practice Research Datalink.

## References

[1] van Riet EES , Hoes AW , Wagenaar KP , et al . Epidemiology of heart failure: the prevalence of heart failure and ventricular dysfunction in older adults over time. A systematic review. Eur J Heart Fail 2016;18:242–52. 10.1002/ejhf.483 26727047

[2] Jones NR , Roalfe AK , Adoki I , et al . Survival of patients with chronic heart failure in the community: a systematic review and meta-analysis. Eur J Heart Fail 2019;21:1306–25. 10.1002/ejhf.1594 31523902 PMC6919428

[3] Taylor CJ , Ordóñez-Mena JM , Roalfe AK , et al . Trends in survival after a diagnosis of heart failure in the United Kingdom 2000-2017: population based cohort study. BMJ 2019;364:l223. 10.1136/bmj.l223 30760447 PMC6372921

[4] World Health Organisation . Obesity and overweight. 2021. Available: https://www.who.int/news-room/fact-sheets/detail/obesity-and-overweight [Accessed 11 Jan 2022].

[5] Hu G , Jousilahti P , Antikainen R , et al . Joint effects of physical activity, body mass index, waist circumference, and waist-to-hip ratio on the risk of heart failure. Circulation 2010;121:237–44. 10.1161/CIRCULATIONAHA.109.887893 20048205

[6] Mahajan R , Stokes M , Elliott A , et al . Complex interaction of obesity, intentional weight loss and heart failure: a systematic review and meta-analysis. Heart 2020;106:58–68. 10.1136/heartjnl-2019-314770 31530572

[7] Loehr LR , Rosamond WD , Poole C , et al . Association of multiple Anthropometrics of overweight and obesity with incident heart failure: the Atherosclerosis risk in communities study. Circ Heart Fail 2009;2:18–24. 10.1161/CIRCHEARTFAILURE.108.813782 19808311 PMC2748859

[8] Lavie CJ , Alpert MA , Arena R , et al . Impact of obesity and the obesity paradox on prevalence and prognosis in heart failure. JACC Heart Fail 2013;1:93–102. 10.1016/j.jchf.2013.01.006 24621833

[9] Vergaro G , Gentile F , Meems LMG , et al . NT-proBNP for risk prediction in heart failure: identification of optimal cutoffs across body mass index categories. JACC Heart Fail 2021;9:653–63. 10.1016/j.jchf.2021.05.014 34246607

[10] Obesity: preventing and managing the global epidemic. report of a WHO consultation. World Health Organ Tech Rep Ser 2000;894:i–xii. 11234459

[11] Caleyachetty R , Barber TM , Mohammed NI , et al . Ethnicity-specific BMI cutoffs for obesity based on type 2 diabetes risk in England: a population-based cohort study. Lancet Diabetes Endocrinol 2021;9:419–26. 10.1016/S2213-8587(21)00088-7 33989535 PMC8208895

[12] Sterne JAC , White IR , Carlin JB , et al . Multiple imputation for missing data in Epidemiological and clinical research: potential and pitfalls. BMJ 2009;338:b2393. 10.1136/bmj.b2393 19564179 PMC2714692

[13] Therneau TM . A package for survival analysis in R. 2022. Available: https://CRAN.R-project.org/package=survival

[14] Kassambara A , Kosinski M , Biecek P . Drawing survival curves using 'ggplot2' 2021. Available: https://CRAN.R-project.org/package=survminer

[15] Oga EA , Eseyin OR . The obesity paradox and heart failure: a systematic review of a decade of evidence. J Obes 2016;2016:9040248. 10.1155/2016/9040248 26904277 PMC4745816

[16] Sharma A , Lavie CJ , Borer JS , et al . Meta-analysis of the relation of body mass index to all-cause and cardiovascular mortality and hospitalization in patients with chronic heart failure. Am J Cardiol 2015;115:1428–34. 10.1016/j.amjcard.2015.02.024 25772740

[17] Zhang J , Begley A , Jackson R , et al . Body mass index and all-cause mortality in heart failure patients with normal and reduced ventricular ejection fraction: a dose-response meta-analysis. Clin Res Cardiol 2019;108:119–32. 10.1007/s00392-018-1302-7 29951802

[18] Marcks N , Aimo A , Januzzi JL , et al . Re-appraisal of the obesity paradox in heart failure: a meta-analysis of individual data. Clin Res Cardiol 2021;110:1280–91. 10.1007/s00392-021-01822-1 33704552 PMC8318940

[19] Nicholson BD , Aveyard P , Bankhead CR , et al . Determinants and extent of weight recording in UK primary care: an analysis of 5 million adults' electronic health records from 2000 to 2017. BMC Med 2019;17:222. 10.1186/s12916-019-1446-y 31783757 PMC6883613

[20] Iliodromiti S , Celis-Morales CA , Lyall DM , et al . The impact of confounding on the associations of different Adiposity measures with the incidence of cardiovascular disease: a cohort study of 296 535 adults of white European descent. Eur Heart J 2018;39:1514–20. 10.1093/eurheartj/ehy057 29718151 PMC5930252

[21] Powell-Wiley TM , Poirier P , Burke LE , et al . Obesity and cardiovascular disease: a scientific statement from the American Heart Association. Circulation 2021;143:e984–1010. 10.1161/CIR.0000000000000973 33882682 PMC8493650

[22] Oktay AA , Lavie CJ , Kokkinos PF , et al . The interaction of cardiorespiratory fitness with obesity and the obesity paradox in cardiovascular disease. Prog Cardiovasc Dis 2017;60:30–44. 10.1016/j.pcad.2017.05.005 28502849

[23] Bhatt AS , Cooper LB , Ambrosy AP , et al . Interaction of body mass index on the association between N-terminal-pro-B-type natriuretic peptide and morbidity and mortality in patients with acute heart failure: findings from ASCEND-HF (acute study of clinical effectiveness of Nesiritide in decompensated heart failure). J Am Heart Assoc 2018;7:e006740. 10.1161/JAHA.117.006740 29431103 PMC5850232

[24] Kenchaiah S , Pocock SJ , Wang D , et al . Body mass index and prognosis in patients with chronic heart failure: insights from the Candesartan in heart failure: assessment of reduction in mortality and morbidity (CHARM) program. Circulation 2007;116:627–36. 10.1161/CIRCULATIONAHA.106.679779 17638930

[25] Sharma K , Mok Y , Kwak L , et al . Predictors of mortality by sex and race in heart failure with preserved ejection fraction: ARIC community surveillance study. JAHA 2020;9:19. 10.1161/JAHA.119.014669 PMC7792380 32924735

[26] Clark AL , Fonarow GC , Horwich TB . Impact of cardiorespiratory fitness on the obesity paradox in patients with systolic heart failure. Am J Cardiol 2015;115:209–13. 10.1016/j.amjcard.2014.10.023 25465933

[27] Elagizi A , Carbone S , Lavie CJ , et al . Implications of obesity across the heart failure continuum. Prog Cardiovasc Dis 2020;63:561–9. 10.1016/j.pcad.2020.09.005 33002458 PMC7521376

[28] El Hajj EC , El Hajj MC , Sykes B , et al . Pragmatic weight management program for patients with obesity and heart failure with preserved ejection fraction. J Am Heart Assoc 2021;10:e022930. 10.1161/JAHA.121.022930 34713711 PMC8751835

[29] Mandviwala TM , Basra SS , Khalid U , et al . Obesity and the paradox of mortality and heart failure hospitalization in heart failure with preserved ejection fraction. Int J Obes (Lond) 2020;44:1561–7. 10.1038/s41366-020-0563-1 32483205

[30] Pandey A , Kitzman D , Reeves G . Frailty is intertwined with heart failure: mechanisms, prevalence, prognosis, assessment, and management. JACC Heart Fail 2019;7:1001–11. 10.1016/j.jchf.2019.10.005 31779921 PMC7098068

