# Supplementary material for: Body mass index and survival in people with heart failure
Source: Heart. 2023 Jun 8;109(20):1542–9. doi: 10.1136/heartjnl-2023-322459 (PMC10579501; doi:10.1136/heartjnl-2023-322459)
Supplement: Supplementary data [file heartjnl-2023-322459supp001.pdf]

Supplemental Table 1 Baseline characteristics of people with heart failure and available or missing BMI measurement.

**Variable**	**With BMI (n=47531)**		**Without BMI (n=8428)**	
**Gender, n (%)**				
Men	25755	(54.19)	3479	(41.28)
Women	21776	(45.81)	4949	(58.72)
**Age (years), median (IQR)**	78	(70 - 84)	82	(74 - 88)
**Age category, n (%)**				
45-54	1664	(3.50)	274	(3.25)
55-64	4809	(10.12)	617	(7.32)
65-74	11189	(23.54)	1296	(15.38)
75-84	18596	(39.12)	2938	(34.86)
85-94	10574	(22.25)	2879	(34.16)
94+	699	(1.47)	424	(5.03)
**Ethnicity, n (%)**				
White	37537	(78.97)	6606	(78.38)
Non-white	1348	(2.84)	149	(1.77)
Mixed	6078	(12.79)	507	(6.02)
Missing	2568	(5.40)	1166	(13.83)
**Smoking Status, n (%)**				
Never	18106	(38.09)	3146	(37.33)
Former	6152	(12.94)	942	(11.18)
Current	22751	(47.87)	1756	(20.84)
Missing	522	(1.10)	2584	(30.66)
**Index of multiple deprivation (quintile), n (%)**				
1 (most deprived)	9244	(19.45)	1610	(19.10)
2	10854	(22.84)	2100	(24.92)
3	10177	(21.41)	1770	(21.00)
4	10038	(21.12)	1669	(19.80)
5 (least deprived)	7175	(15.10)	1272	(15.09)
Missing	43	(0.09)	7	(0.08)
**Medical history of**				
Atrial Fibrillation, n (%)	12980	(27.31)	1649	(19.57)
Angina, n (%)	10871	(22.87)	1094	(12.98)
Diabetes, n (%)	12425	(26.14)	679	(8.06)
Hypertension, n (%)	29052	(61.12)	3264	(38.73)
Ischaemic Heart Disease, n (%)	13196	(27.76)	1410	(16.73)
Myocardial infarction, n (%)	10271	(21.61)	1025	(12.16)
Other Cardiovascular disease, n (%)	12166	(25.60)	1591	(18.88)
Stroke, n (%)	5432	(11.43)	839	(9.95)
Valvular disease, n (%)	3736	(7.86)	418	(4.96)
**BMI (kg/m2)**	27.06	(23.88 - 31)		n/a
**Systolic blood pressure (mmHg)**	137	(123 - 150)	140	(128 - 154)
Missing	212	(0.45)	1060	(12.58)

**Diastolic blood pressure (mmHg)**	78	(70 - 83)	80	(70 - 85)
Missing	212	(0.45)	1060	(12.58)
**Total cholesterol (mmol/L)**	4.5	(3.8 - 5.4)	4.9	(4.1 - 5.8)
Missing	10140	(21.33)	5338	(63.34)

Abbreviations: BMI: body mass index; IQR: interquartile range

Supplemental Table 2 Age-adjusted survival rates at 1,5, 10, and 15 years after a diagnosis of heart failure stratified by BMI category.

**BMI category:**	**Age-adjusted survival rate (% (95% CI))**			
	**1 year**	**5 years**	**10 years**	**15 years**
Underweight	67.26 (65.31 to 69.21)	28.1 (25.52 to 30.68)	7.6 (6.16 to 9.04)	1.42 (0.92 to 1.91)
Healthy weight	77.02 (76.48 to 77.55)	43.35 (42.49 to 44.21)	18.33 (17.51 to 19.15)	6.06 (5.3 to 6.83)
Overweight	80.88 (80.42 to 81.34)	50.7 (49.91 to 51.49)	25.19 (24.3 to 26.07)	10.25 (9.21 to 11.29)
Obesity class I	81.43 (80.86 to 82.01)	51.82 (50.74 to 52.9)	26.33 (25.15 to 27.51)	11.03 (9.82 to 12.24)
Obesity class II	81.6 (80.7 to 82.51)	52.17 (50.37 to 53.97)	26.69 (24.77 to 28.6)	11.28 (9.66 to 12.9)
Obesity class III	78.07 (76.64 to 79.49)	45.27 (42.66 to 47.89)	20.02 (17.63 to 22.4)	7.01 (5.46 to 8.56)

Abbreviations: BMI: body mass index

Supplemental Table 3 Survival rates at 1, 5, 10, and 15 years after a diagnosis of heart failure stratified by age and BMI category

Subgroup	Survival rate (% (95% CI))			
	**At 1 year**	**At 5 years**	**At 10 years**	**At 15 years**
45-54 years	90.3 (88.97 to 91.65)	78.45 (76.36 to 80.6)	64.73 (61.61 to 68.01)	54.44 (49.98 to 59.3)
By BMI category:				
Underweight	87.84 (73.37 to 100)	60.39 (32.56 to 100)	40.26 (14.65 to 100)	NA
Healthy weight	88.67 (85.2 to 92.28)	75.48 (70.23 to 81.11)	61.43 (53.88 to 70.04)	50.46 (40.53 to 62.82)
Overweight	91.53 (89.16 to 93.96)	82.41 (78.82 to 86.16)	69.38 (63.56 to 75.73)	57.22 (48.28 to 67.81)
Obesity class I	91.27 (88.46 to 94.17)	80.89 (76.49 to 85.53)	68.99 (62.28 to 76.42)	63.02 (54.44 to 72.95)
Obesity class II	90.9 (86.97 to 95.01)	76.34 (69.8 to 83.5)	61.99 (52.35 to 73.39)	51.88 (39.79 to 67.65)
Obesity class III	89.71 (85.42 to 94.21)	74.87 (67.48 to 83.08)	57.47 (47.2 to 69.99)	57.47 (47.2 to 69.99)
55-64 years	87.88 (87 to 88.77)	70.63 (69.27 to 72.01)	52.78 (50.93 to 54.7)	38.43 (35.56 to 41.53)
By BMI category:				
Underweight	69.84 (59.34 to 82.18)	47.77 (36.02 to 63.34)	30.98 (18.34 to 52.32)	30.98 (18.34 to 52.32)
Healthy weight	83.94 (81.64 to 86.3)	65.77 (62.54 to 69.16)	49.72 (45.62 to 54.18)	36.59 (30.74 to 43.55)
Overweight	88.82 (87.21 to 90.45)	74.97 (72.54 to 77.49)	56.27 (52.71 to 60.07)	42.09 (36.38 to 48.69)
Obesity class I	90.27 (88.54 to 92.03)	72.79 (69.89 to 75.8)	56.05 (52.12 to 60.29)	45.62 (40.37 to 51.56)
Obesity class II	92.49 (90.38 to 94.65)	73.23 (69.21 to 77.47)	51.09 (45.05 to 57.93)	27.66 (17.74 to 43.14)
Obesity class III	89.26 (86.59 to 92.01)	68.18 (63.64 to 73.05)	49.56 (43.41 to 56.58)	25.84 (17.1 to 39.06)
65-74 years	83.45 (82.79 to 84.12)	59.08 (58.12 to 60.06)	35.38 (34.2 to 36.61)	17.23 (15.48 to 19.17)
By BMI category:				
Underweight	59.68 (52.53 to 67.8)	29.22 (22.18 to 38.51)	21.74 (15.07 to 31.36)	21.74 (15.07 to 31.36)
Healthy weight	78.11 (76.53 to 79.73)	53.29 (51.19 to 55.47)	31.78 (29.33 to 34.43)	15.37 (11.9 to 19.86)
Overweight	85.79 (84.7 to 86.89)	61.49 (59.81 to 63.22)	37.88 (35.8 to 40.09)	16.45 (13.34 to 20.28)
Obesity class I	86.58 (85.26 to 87.93)	62.81 (60.71 to 64.99)	36.43 (33.67 to 39.41)	17.89 (14.43 to 22.19)
Obesity class II	87.32 (85.38 to 89.31)	65.26 (62.15 to 68.52)	38.79 (34.63 to 43.44)	24.89 (18.71 to 33.12)
Obesity class III	85.68 (82.98 to 88.47)	57.37 (52.93 to 62.2)	32.14 (26.31 to 39.25)	13.39 (6.27 to 28.59)
75-84 years	76.54 (75.97 to 77.12)	43.2 (42.43 to 43.97)	18.37 (17.56 to 19.21)	5.83 (4.85 to 7.01)
By BMI category:				
Underweight	59.85 (55.21 to 64.88)	24.7 (20.24 to 30.15)	7.46 (4.06 to 13.68)	NA
Healthy weight	73.04 (71.9 to 74.2)	38.81 (37.39 to 40.28)	15.28 (13.91 to 16.78)	4.8 (3.36 to 6.85)
Overweight	79.61 (78.66 to 80.57)	46.9 (45.57 to 48.27)	20.7 (19.21 to 22.3)	7.3 (5.23 to 10.18)

Obesity class I	81.68 (80.38 to 83)	48.42 (46.48 to 50.44)	21.49 (19.26 to 23.98)	6.91 (4.38 to 10.91)
Obesity class II	81.26 (78.97 to 83.62)	51 (47.6 to 54.64)	24.32 (20.28 to 29.16)	8.24 (3.84 to 17.68)
Obesity class III	82.25 (78.64 to 86.02)	45.25 (39.57 to 51.75)	21.57 (15.33 to 30.34)	NA
85-94 years	63.22 (62.38 to 64.06)	22.26 (21.37 to 23.18)	4.41 (3.76 to 5.18)	0.23 (0.04 to 1.38)
By BMI category:				
Underweight	47.8 (42.89 to 53.27)	13.88 (9.97 to 19.32)	NA	NA
Healthy weight	61.49 (60.06 to 62.97)	20.56 (19.08 to 22.15)	5.03 (3.87 to 6.53)	0.74 (0.15 to 3.77)
Overweight	68.52 (67.02 to 70.04)	25.45 (23.71 to 27.31)	4.95 (3.65 to 6.7)	NA
Obesity class I	69.03 (66.5 to 71.66)	30.48 (27.4 to 33.91)	6.29 (3.89 to 10.17)	NA
Obesity class II	67.43 (61.97 to 73.37)	28.8 (22.43 to 36.96)	6.56 (2.46 to 17.52)	NA
Obesity class III	73.33 (63.35 to 84.87)	15.93 (7.22 to 35.14)	NA	NA
94+ years	43.9 (40.95 to 47.05)	6.03 (4.38 to 8.29)	43.9 (40.95 to 47.05)	6.03 (4.38 to 8.29)
By BMI category:				
Underweight	32.29 (18.85 to 55.33)	NA	NA	NA
Healthy weight	45.56 (40.67 to 51.04)	7.99 (4.95 to 12.91)	NA	NA
Overweight	55.41 (48.59 to 63.19)	7.52 (3.72 to 15.2)	NA	NA
Obesity class I	47.76 (35.67 to 63.95)	11.17 (3.78 to 33.06)	NA	NA
Obesity class II	43.96 (23.28 to 83.01)	NA	NA	NA
Obesity class III	NA	NA	NA	NA

Abbreviations: BMI: body mass index; N: not available (insufficient data for this analysis)

Supplemental Table 4 Adjusted hazard Ratios (HR), 95% Confidence Intervals (CI) and the risk difference between BMI categories with all-cause mortality among people with heart failure.*

BMI Category	Adjusted HR	Absolute Risk	Risk Difference
Healthy weight (reference)	1.00	18.8% (8,959/14,880*100)	-
Overweight	0.78	18.8% * 0.78 = 14.7%	-4.1%
Obesity class I	0.76	18.8% * 0.76 = 14.3%	-4.5%
Obesity class II	0.76	18.8% * 0.76 = 14.3%	-4.5%
Obesity class III	0.96	18.8% * 0.96 = 18.1%	-0.7%
Underweight	1.59	18.8% * 1.59 = 30.0%	11.2%

Among participants with Heart Failure and healthy weight (n = 14,880), 18.8% (n = 8,959) died during follow-up. Risk differences calculated using the adjusted hazard ratios reported in Table 3.

* Adjusted for age, smoking status, index of multiple deprivation, ethnicity, systolic and diastolic blood pressure, total cholesterol, and history of atrial fibrillation, angina, diabetes, hypertension, ischaemic heart disease, myocardial infarction, stroke, valvular heart disease, and other cardiovascular diseases.

Supplemental Table 5 Hazard Ratios (HR) and 95% Confidence Intervals (CI) for the association of BMI categories with all-cause mortality among men and women with heart failure.*

BMI	Men				Women				P-value
**Category**	**N**	**n**	**HR**	**(95% CI)**	**N**	**n**	**HR**	**(95% CI)**	**interaction**
Underweight (<18.5 kg/m^2)	209	149	1.90	(1.61 to 2.24)	472	297	1.48	(1.31 to 1.67)	0.02
Healthy weight (18.5-25 kg/m^2)	5340	2998	1.00	(Reference)	4883	2581	1.00	(Reference)	-
Overweight (25-30 kg/m^2)	8255	3855	0.77	(0.73 to 0.81)	4816	2199	0.79	(0.75 to 0.84)	0.42
Obesity class I (30-35 kg/m^2)	4247	1789	0.76	(0.71 to 0.81)	2988	1256	0.77	(0.72 to 0.83)	0.57
Obesity class II (35-40 kg/m^2)	1490	569	0.77	(0.7 to 0.85)	1414	554	0.75	(0.68 to 0.82)	0.72
Obesity class III (≥40 kg/m^2)	676	216	0.91	(0.79 to 1.05)	938	378	0.97	(0.87 to 1.09)	0.31

Abbreviations: **N**: number at risk of dying within the relevant BMI category; **n**: number dying from all-causes for the BMI category.

* Adjusted for age, smoking status, index of multiple deprivation, ethnicity, systolic and diastolic blood pressure, total cholesterol, and history of atrial fibrillation, angina, diabetes, hypertension, ischaemic heart disease, myocardial infarction, stroke, valvular heart disease, and other cardiovascular diseases.

Supplemental Table 6 Hazard Ratios (HR) and 95% Confidence Intervals (CI) for the association of BMI categories with all-cause mortality among people with heart failure and white, non-white or mixed ethnicity.

	White			Non-white				Mixed			
**Category**	**N**	**n**	**HR (95% CI)**	**N**	**n**	**HR (95% CI)**	**p***	**N**	**n**	**HR (95% CI)**	**p***
Underweight	565	379	**1.57 (1.41 to 1.75)**	12	5	1.59 (0.64 to 3.95)	0.83	104	62	**1.76 (1.35 to 2.29)**	0.51
Healthy weight	8416	4763	1.00 (Reference)	331	143	1.00 (Reference)	-	1476	673	1.00 (Reference)	-
Overweight	10700	5211	**0.78 (0.75 to 0.81)**	429	170	0.89 (0.71 to 1.11)	0.32	1942	673	**0.75 (0.67 to 0.83)**	0.20
Obesity class I	5799	2552	**0.75 (0.72 to 0.79)**	273	88	**0.68 (0.51 to 0.89)**	0.56	1163	405	**0.87 (0.76 to 0.99)**	0.56
Obesity class II	2340	934	**0.73 (0.67 to 0.78)**	103	39	1.10 (0.76 to 1.59)	0.03	461	150	1.02 (0.85 to 1.23)	0.12
Obesity class III	1306	514	0.94 (0.85 to 1.03)	51	14	0.64 (0.36 to 1.14)	0.52	257	66	1.11 (0.86 to 1.45)	0.61

Abbreviations: BMI: body mass index; HR: hazard ratio; N: number at risk of dying from CVD within the relevant BMI category; n: number dying from CVD for the BMI category.

* p-value for interaction

Supplemental Table 7 Sensitivity analysis comparing different approaches to handling missing data in the primary analysis, in which we report the Hazard Ratios (HR) and 95% Confidence Intervals (CI) for the association of BMI categories with all-cause mortality among people with heart failure.

	**Adjusted HR (95% CI)**			
**Category**	**Complete case analysis (n = 35,728)** [a]	**Missing indicator analysis (n = 37,278)** [b]	**Excluding total cholesterol (n = 44,280)** [c]	**Multiple imputation (n = 47,531)** [d]
**Underweight**	**1.59 (1.45 to 1.75)**	**1.57 (1.43 to 1.73)**	**1.56 (1.44 to 1.68)**	**1.51 (1.40 to 1.63)**
**Healthy weight**	1.00 (Reference)	1.00 (Reference)	1.00 (Reference)	1.00 (Reference)
**Overweight**	**0.78 (0.75 to 0.81)**	**0.79 (0.76 to 0.82)**	**0.80 (0.77 to 0.82)**	**0.79 (0.77 to 0.82)**
**Obesity class I**	**0.76 (0.73 to 0.80)**	**0.77 (0.74 to 0.81)**	**0.77 (0.74 to 0.81)**	**0.77 (0.75 to 0.80)**
**Obesity class II**	**0.76 (0.71 to 0.81)**	**0.77 (0.72 to 0.82)**	**0.77 (0.73 to 0.82)**	**0.76 (0.72 to 0.81)**
**Obesity class III**	0.96 (0.88 to 1.05)	0.97 (0.89 to 1.06)	0.96 (0.89 to 1.04)	0.95 (0.88 to 1.03)

Abbreviations: **n**: number of participants included in analysis.

[a] Excluded those with missing information on ethnicity, smoking status, index of multiple deprivation, systolic and diastolic blood pressure, and total cholesterol.

[b] Excluded those with missing data on systolic and diastolic blood pressure, and total cholesterol, and added missing indicator variables for smoking, ethnicity, and index of multiple deprivation.

[c] Did not adjust for total cholesterol, all other covariates were kept the same. Those with missing data on ethnicity, smoking status, index of multiple deprivation, systolic and diastolic blood pressure were excluded.

[d] Adjusted for the same set of variables as in the complete case analyses. Results were pooled across 5 imputed datasets.

Supplemental Table 8 Hazard ratios and 95% confidence intervals for the association of BMI categories with all-cause mortality among people with heart failure after excluding those dying within 1, 2, 3, 4, and 5 years after the assessment of BMI.*

**Time between BMI assessment and death**	**Underweight (<18.5 kg/m^2)**	**Healthy weight (18.5-25 kg/m^2)**	**Overweight (25-30 kg/m^2)**	**Obesity class I (30-35 kg/m^2)**	**Obesity class II (35-40 kg/m^2)**	**Obesity class III (≥40 kg/m^2)**
**≥ 1 year**	331/171, **1.38 (1.19 to 1.62)**	6482/2986, 1.00 (Reference)	9403/3782, **0.82 (0.78 to 0.86)**	5324/1981, **0.83 (0.78 to 0.88)**	2170/734, **0.82 (0.75 to 0.89)**	1188/392, 1.10 (0.98 to 1.23)
**≥ 2 year**	223/112, **1.41 (1.16 to 1.71)**	4771/2098, 1.00 (Reference)	7410/2887, **0.85 (0.81 to 0.90)**	4189/1497, **0.85 (0.79 to 0.91)**	1741/580, **0.87 (0.79 to 0.96)**	916/292, **1.14 (1.00 to 1.30)**
**≥ 3 year**	151/67, 1.27 (0.99 to 1.62)	3548/1510, 1.00 (Reference)	5739/2158, **0.86 (0.81 to 0.92)**	3302/1161, **0.88 (0.81 to 0.95)**	1368/437, **0.87 (0.78 to 0.97)**	685/216, 1.14 (0.98 to 1.33)
**≥ 4 year**	105/45, 1.27 (0.94 to 1.72)	2640/1086, 1.00 (Reference)	4439/1617, **0.88 (0.81 to 0.95)**	2548/854, **0.88 (0.80 to 0.97)**	1084/339, 0.91 (0.80 to 1.03)	531/158, 1.12 (0.94 to 1.34)
**≥ 5 year**	70/26, 1.09 (0.74 to 1.61)	1959/781, 1.00 (Reference)	3377/1186, **0.86 (0.79 to 0.95)**	1982/644, 0.89 (0.80 to 1.00)	830/261, 0.93 (0.80 to 1.08)	407/113, 1.08 (0.88 to 1.33)

Abbreviations: BMI: body mass index

* The numbers shown in each cell correspond to the number at risk, number dying, the hazard ratio and its confidence intervals within brackets.

Supplemental Table 9 Numbers and causes of death of people with heart failure stratified by BMI category.

**Causes of death subgroup, n (%)**	**Underweight (<18.5 kg/m²)**		**Healthy weight (18.5-25 kg/m²)**		**Overweight (25-30 kg/m²)**		**Obesity class I (30-35 kg/m²)**		**Obesity class II (35-40 kg/m²)**		**Obesity class III (≥40 kg/m²)**	
Total Number of Deaths	756	(100)	8959	(100)	8939	(100)	4197	(100)	1417	(100)	745	(100)
Death due to cardiovascular disease	383	(50.7)	4955	(55.3)	5008	(56.0)	2338	(55.7)	826	(58.3)	403	(54.1)
Heart failure primary cause	63	(8.33)	675	(7.53)	533	(5.96)	278	(6.62)	86	(6.07)	65	(8.72)
Heart failure any cause of death*	347	(45.9)	3891	(43.4)	3659	(40.9)	1774	(42.3)	623	(44.0)	348	(46.7)
Arrhythmias (ICD-10 codes: I47-	20	(2.65)	162	(1.81)	136	(1.52)	59	(1.41)	24	(1.69)	7	(0.94)
Diseases of the respiratory system	196	(25.9)	1526	(17.0)	1334	(14.9)	573	(13.7)	184	(13.0)	111	(14.9)
Cancer	59	(7.80)	1061	(11.8)	1243	(13.9)	615	(14.7)	179	(12.6)	72	(9.66)
Diseases of the digestive system	32	(4.23)	295	(3.29)	333	(3.73)	138	(3.29)	62	(4.38)	33	(4.43)
Diseases of the genitourinary	19	(2.51)	197	(2.20)	222	(2.48)	110	(2.62)	40	(2.82)	24	(3.22)
Other	67	(8.86)	925	(10.3)	799	(8.94)	423	(10.1)	126	(8.89)	102	(13.7)

* Includes patients for whom heart failure may have been the primary or a contributory cause of death.

Supplemental Table 10 Hazard ratios and 95% confidence intervals for the association of BMI categories with CVD mortality among people with heart failure.

BMI Category	N	n	Crude model		Age & Sex adjusted		N	n	Multivariable model	
Underweight (<18.5 kg/m^2)	1084	383	1.41	(1.27 to 1.57)	1.42	(1.28 to 1.57)	681	236	1.51	(1.32 to 1.73)
Healthy weight (18.5-25 kg/m^2)	14880	4955	1.00	(Reference)	1.00	(Reference)	10223	3096	1.00	(Reference)
Overweight (25-30 kg/m^2)	17277	5008	0.76	(0.73 to 0.79)	0.82	(0.79 to 0.85)	13071	3387	0.79	(0.75 to 0.83)
Obesity class I (30-35 kg/m^2)	9013	2338	0.65	(0.62 to 0.69)	0.80	(0.76 to 0.84)	7235	1681	0.77	(0.72 to 0.81)
Obesity class II (35-40 kg/m^2)	3400	826	0.60	(0.56 to 0.64)	0.85	(0.78 to 0.91)	2904	630	0.78	(0.71 to 0.85)
Obesity class III (≥40 kg/m^2)	1877	403	0.55	(0.50 to 0.61)	0.97	(0.87 to 1.08)	1614	329	0.97	(0.86 to 1.09)

Abbreviations: BMI: body mass index; N: number at risk of dying from CVD within the relevant BMI category; n: number dying from CVD for the BMI category.

* The numbers at risk and dying from CVD are smaller in the multivariable model due to missing data.

** Adjusted for sex, age, smoking status, index of multiple deprivation, ethnicity, systolic and diastolic blood pressure, total cholesterol, and history of atrial fibrillation, angina, diabetes, hypertension, ischaemic heart disease, myocardial infarction, stroke, valvular heart disease, and other cardiovascular diseases.
